# Supplementary material for: Alpine-style nappes thrust over ancient North China continental margin demonstrate large Archean horizontal plate motions
Source: Nat Commun. 2021 Oct 26;12:6172. doi: 10.1038/s41467-021-26474-7 (PMC8548327; doi:10.1038/s41467-021-26474-7)
Supplement: Supplementary file 1 — Supplementary Information [file 41467_2021_26474_MOESM1_ESM.pdf]

# Supplementary Information

## Supplementary Note 1

## Geochronology of the Zanhuang fold nappe units

### Zircon U-Pb Data

Four samples from the Zanhuang fold nappe/thrust belt were selected for LA-ICP-MS zircon U–Pb dating, including tholeiitic metabasalts (19NQ-2 and 18Bd-13) from Buddha nappe, a metamorphic felsic dike (19ZY-2) crosscutting picritic-boninitic metabasite from Black Rock Temple nappe, and *schistes lustrés* (19SL). The CL images of representative zircon grains are illustrated with the zircon U–Pb isotope data with Rare Earth element data that are plotted in Fig. S1, S2 and listed in Supplementary data 1. GPS locations of samples are given in Fig. S3.

***Tholeiitic metabasalts***

All zircons are subhedral to euhedral, colorless or transparent under the optical microscope, and have grain sizes of 50–150 $\mu m$ with length-to-width ratios of 1:1 to 2:1. The majority of zircons from both samples (19NQ-2 and 18Bd-13) show oscillatory or weak oscillatory zoning, characterized by dull luminescence, with or without narrow bright rims, indicating that they are likely magmatic zircons with varying degrees of metamorphic overgrowth[1] (Fig. S1a, c). The minor, metamorphic zircons, in sample 19NQ-2 and 18Bd-13 show euherdral to subhedral shapes with varying grain sizes of 50–100 $\mu m$. The cathodoluminescence (CL) images reveal cores with blurred irregular banded/sector-zoning, or fir-tree zoning that are commonly considered to be metamorphic origin[2-6] (Fig. S1c).

Thirteen analyses on domains with oscillatory zoning from sample 19NQ-2 (e.g., spots 07 and 11 in Fig. S1a) yielded a small range of Th/U values (0.26–0.88). The REE patterns show positive Ce anomalies, moderate negative Eu anomalies and steep HREE patterns (Fig. S2a), indicative of their magmatic origin[5,7]. These analyses on U-Pb concordia diagrams show linear arrays and yielded upper intercept ages of 2713±33 Ma (n=13; MSWD=0.20), which is consistent with weighted mean $^{207}Pb/^{206}Pb$ age, 2698±30 Ma (MSWD=0.15) of 5 concordant ages varying from 2682±34 Ma and 2718±34 Ma (i.e., spots 02, 03, 04, 07 and 11 in

Supplementary data 1) within error. Thus the 2698±30 Ma is interpreted as the crystallization age of igneous zircons in sample 19NQ-2.

Nine analyses on the bright rim domains and metamorphic origin zircons give Th/U values of 0.002–0.01, and the U content varies from 78 to 584 ppm. Most of the analyses are concordant and yield an upper intercept age of 1852±29 Ma (n=9; MSWD = 0.37) and 8 concordant data give a weighted mean $^{207}Pb/^{206}Pb$ age of 1852±38 Ma (MSWD = 0.36). The analyses exhibit steep HREE patterns with no Eu anomalies or slightly positive Eu anomalies, which suggests limited growth of the Eu compatible mineral plagioclase, with no significant concurrent growth of HREE compatible mineral garnet during the circa 1852 Ma metamorphism[3,8].

Nineteen out of twenty-one analyses are on the oscillatory zoned domains from sample 18Bd-13 (e.g., spots 03 and 05 in Fig. S1c), which give Th/U values of 0.20–0.69 (only one exception of 0.11) with positive Ce anomalies, moderate negative Eu anomalies and steep HREE patterns, suggesting their magmatic origin[5,7] (Fig. S2b). They show discordant but form a well-correlated linear array and yielded an upper intercept age of 2 699±24 Ma (MSWD=0.30) (Fig. 5c), which agrees perfectly with its weighted mean $^{207}Pb/^{206}Pb$ age, 2701±43 Ma (MSWD=0.14) of 3 concordant ages varying from 2691±37 Ma and 2716±35 Ma (i.e., spots 03, 05 and 07 in Supplementary data 1) within error. The age of 2699 ± 24 Ma is thus viewed as the timing of the igneous zircon crystallized in sample 18Bd-13 meta-basalt rock. The two other analyses on the blurred irregular banded/sector-zoned domains (e.g., spots 06 in Fig. S1c) are all plot on concordia line in the U-Pb concordia diagram. They show $^{207}Pb/^{206}Pb$ concordant age of 2473±45 Ma and 2436±41 Ma, separately, which yield a weighted mean age of 2453±45 Ma that are considered as the timing of following metamorphic event. Their chondrite normalized rare earth elements (REEs) patterns exhibit flat HREE patterns with no significant Eu anomalies, which might indicate the concurrent growth of HREE compatible mineral garnet during the rim domains formation, whereas the partially activated or unstable Eu compatible mineral plagioclase had limited growth during 2453 Ma metamorphism[3,8].

***Felsic dike***

Zircon grains from samples 19ZY-2 mainly exhibit prismatic shapes between 80 and 150 $\mu m$ in length and show colorless or transparent under the optical microscope. In cathodoluminescence (CL) images, the zircon shows intermediate grey fluorescing in CL

emission, are euhedral or subhedral in shape, and have clear and fine oscillatory zoning (Fig. S1d); darker, structureless rims are present in some cases, and represent thin metamorphic overgrowths.

Sixteen analyses on oscillatory zoned domains from sample 19ZY-2 (e.g., spots 01 and 03, 06 in Fig. 4c) yielded Th/U values are of 0.27–0.72 (with only one exception of 0.04) and suggest their magmatic origin, which is compatible with their REE features that exhibit positive Ce anomalies, moderate negative Eu anomalies and steep HREE patterns[5,7] (Fig. S2c). 16 analyses show different degrees of radiogenic Pb-loss but define a well-correlated discordia line with an upper intercept age of 2656±29 Ma (MSWD=1.3) (Fig. S1d), consistent with its $^{207}Pb/^{206}Pb$ concordant age of 2692±35 Ma within error. Therefore, the 2692±35 Ma is interpreted as the timing of crystallization age of the zircons.

***Schistes lustrés***

Most of the examined zircon grains in sample 19SL show an isometric or stubby crystal habit varying from 80 $\mu m$ to 200 $\mu m$ with core-rim structures in cathodoluminescence (CL) images. They are dominated by relict cores with rounded to stubby shape and oscillatory zoning with overgrowth rims that vary in diameter and interior texture (Fig. 6e). The overgrowth rims commonly show stubby and isometric habits but are otherwise featurelessness in CL images (Fig. 6f). Some zircons occur as small anhedral grains without relict oscillatory zoned core domains, usually preserve isometric shape with relatively weaker CL fluorescence. They display blurred sector-zoning, or appear structureless, features that are commonly considered to be of metamorphic origin[2-6] (Fig. 6f).

From thirty analyses on relict oscillatory zoned cores, $^{207}Pb/^{206}Pb$ ages are distributed widely from 2807 to 2524 Ma. The age spectrum is dominated by two main populations at 2780–2620 Ma and 2590–2520 Ma with peaks at ~2700 Ma and 2560 Ma (Fig. S1e). The oldest Meso-Archean zircon grains have ages of 2807±55 Ma, and the 3 youngest zircons yielded ages from 2545±40 Ma to 2524±40 Ma with a weighted mean age of 2536±43 Ma (Fig. S1e). High Th/U ratios of 0.32–0.63 with U concentration scattering from 261 to 2488 ppm (Supplementary data 1) indicates that relict zircons are magmatic and inherited from crustal protoliths, which is also substantiated by their positive Ce anomalies, moderate negative Eu anomalies and steep HREE patterns[5,7] (Fig. S2d). The rounded terminations with internal oscillatory zoning of relict cores and their wide range of concordant $^{207}Pb/^{206}Pb$ ages

spectrum reflect the detrital origin. Therefore, the age yielded from youngest zircon group can be viewed as the maximum age of deposition.

Seven out of thirteen metamorphic analyses on the blurred sector-zoning or structure-less, dark, small anhedral grains are concordant and yielded a weighted mean $^{207}Pb/^{206}Pb$ age of 2455 ±26 Ma (MSWD = 0.1), which is consistent with the upper intercept age of 2471±23 Ma (MSWD=0.25) that yielded by a well-correlated linear array formed by the whole thirteen analyses (Fig. S1f). Therefore, the age of 2455±26 Ma is interpreted as the timing of recrystallization age of the metamorphic zircons during following metamorphic event. The rare earth elements (REEs) of metamorphic recrystallization zircons show elevated LREE and steep HREE patterns with moderately positive or no Eu anomalies which might suggest a significant breakdown of LREE bearing minerals (e.g., monazite, allanite) and limited crystallization or even breakdown of Eu compatible mineral plagioclase, contemporaneously with zircon growth during metamorphism period[3,8].

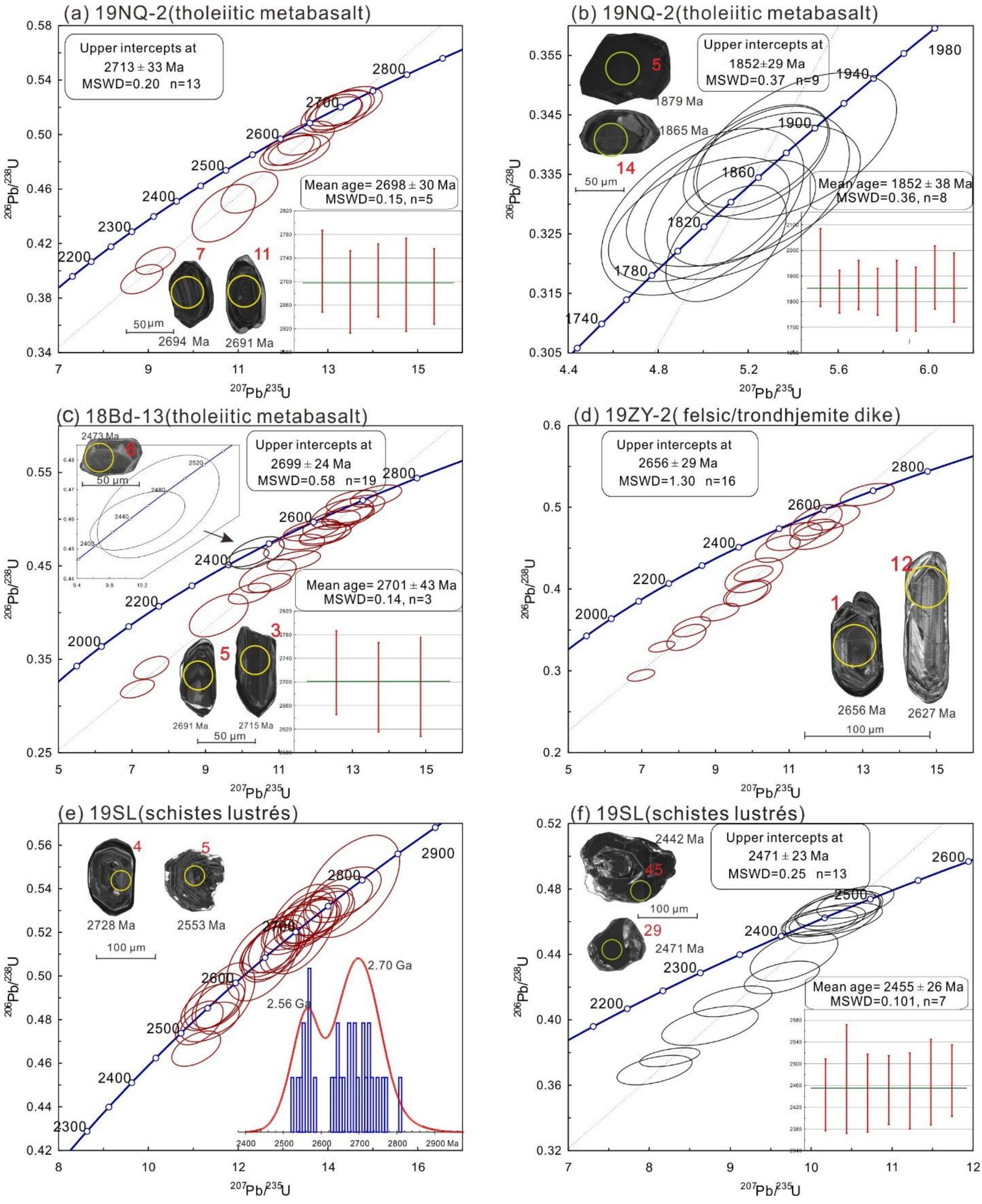


**Fig. S1** U-Pb concordia diagrams and CL images of representative zircon grains showing internal structures, analyzed locations, and calculated apparent $^{207}Pb/^{206}Pb$ ages (Ma). Spot numbers are consistent with those listed in Supplementary data 1. (a) magmatic zircons from tholeiitic metabasalt (19NQ-2); (b) metamorphic zircons from tholeiitic metabasalt (19NQ-2); (c) magmatic zircons from tholeiitic metabasalt (18BD-13); (d) magmatic zircon from felsic/trondhjemite dike (19ZY-2) which crosscut boninites; (e) detrital zircons from *schistés lustrés* (19SL); (f) metamorphic zircon overgrowth from *schistés lustrés* (19SL).

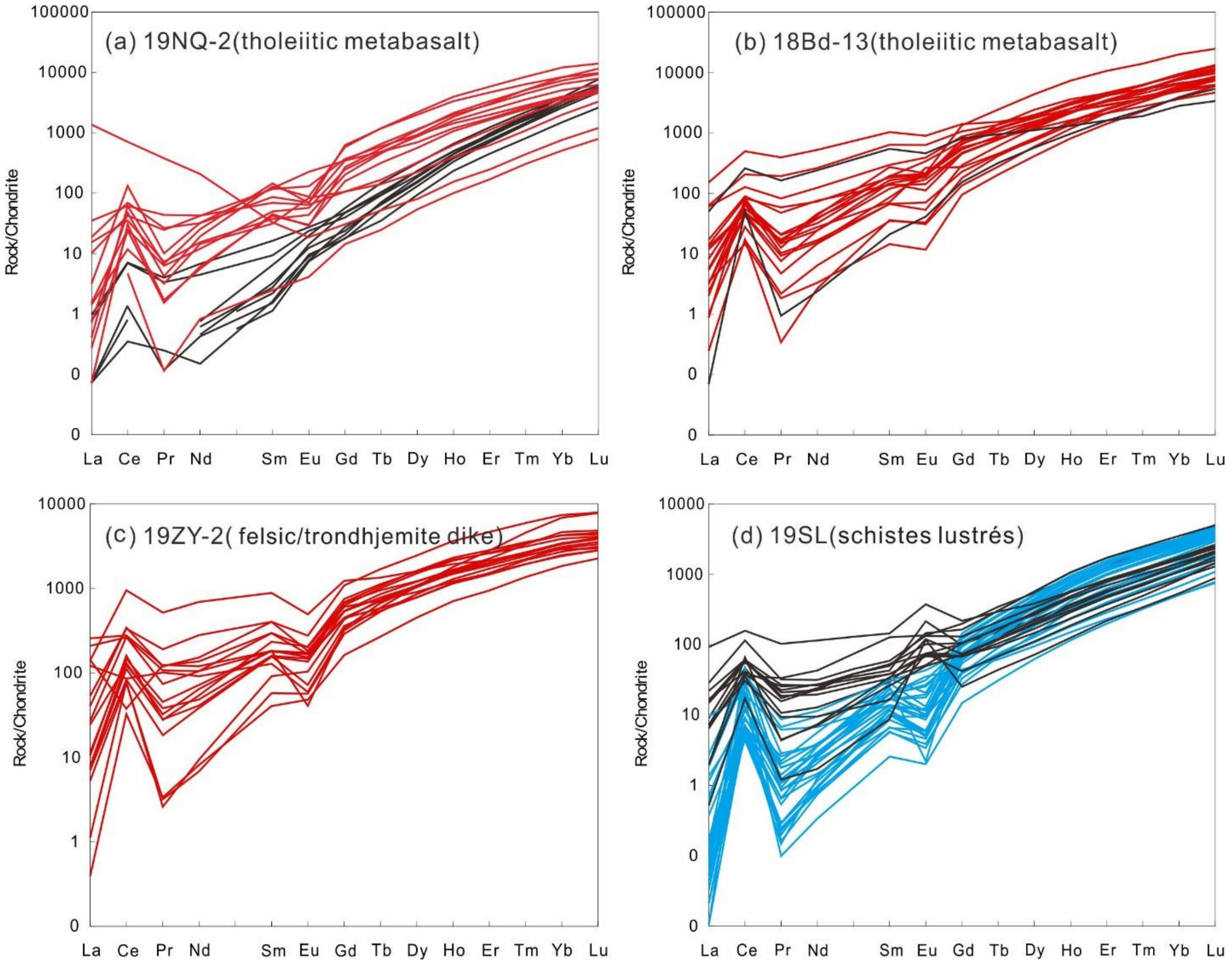


**Fig. S2** Chondrite-normalized REE patterns of the zircons from (a-b) tholeiitic metabasalts; (c) felsic/trondhjemite dike; (d) *schistes lustrés*; the red lines represent magmatic zircons from tholeiitic metabasalts and felsic/trondhjemite dike, the blue lines represent detrital zircons from schistés lustrés; the black lines represent zircons of metamorphic genesis.

GPS LOCATIONS OF SAMPLES

| sample | location | |
|---|---|---|
| | Longitude (East) | Latitude (North) |
| 19NQ-2 | 114° 15′ 36″ | 37° 20′ 25.11″ |
| 18Bd-3 | 114° 15′ 51″ | 37° 20′ 25.39″ |
| 19ZY-2 | 114° 16′ 0.78″ | 37° 18′ 47.91″ |
| 19SL | 114° 15′ 54″ | 37° 20′ 25.31″ |

**Fig. S3.** Location of samples dated in this study.

**Supplementary Note 2.**

**Assessment of Element Mobility During Deformation and Metamorphism**

In deformed and metamorphosed rocks such as those analyzed from the Zanhuang Complex, it is essential to take the element mobility into account before addressing any petrogenetic question, especially samples from the high-strain domains that underwent multiple generations of deformation and metamorphism. First, some elements (e.g., LILE, Rb, Cs Ba and Sr) might become remobilized during post-magmatic alteration[9]. Nevertheless, the low LOI values varying from 0.57 to 1.33 for 17 samples and Ce/Ce* ratios ranging from 0.97 to 1.13 indicate no significant hydration or carbonation and minor LREE mobility[10] (Supplementary data 2), so this issue is of minor concern. Second, the normally immobile high field strength elements (Zr, Ti, Nb, Ta, Hf, Y, Th) and rare earth elements (REE), on which the effect of alteration are proved to be minor in many Archean volcanic rocks[10], show good correlation with the least mobile element Zr on multi-element binary diagrams (Fig. S4a-h), indicating no significant mobility of those elements during the widespread greenschist to amphibolite-facies metamorphic overprint in the study area. However, thorium in some samples shows low degrees of correlation with Zr and a relatively less consistent distribution pattern on chondrite- and primitive mantle-normalized diagrams, suggesting that Th has been, to some extent, affected by alteration, which might be a result of the high extent of the shearing that has been demonstrated in our mapping in the sample area. This is further illustrated on the trace element variation diagrams in Fig. 7d, f in text. Therefore, the aforementioned relatively immobile elements were employed for following discussions of petrogenesis and tectonic implications, while thorium should be used with caution.

## Assessment of Contamination by Continental Crust

Mafic magmas are mantle derived and may be erupted or intruded through continental crust, older arc crust, or represent primary melts from the mantle that did not interact with or assimilate older material, as in oceanic and immature forearc settings. If the magmas pass through continental or arc crust, they usually chemically interact to some extent with the continental crust during ascent through the crust and/or residence in crustal magma chambers. Several lines of geological evidence indicate precursor magmas of the amphibolites show negligible crustal assimilation: (1) the absence of crustal xenolith (e.g. continental detritus) and xenocrystic zircon in the metavolcanic amphibolites and the selected amphibolitic samples, respectively; the felsic dike is younger than the metabasite rocks; (2) high degree of contamination by crustal materials would induce a sharp increase in contents of the elements concentrated in the upper continental crust (e.g. Zr, Hf and LREE) and a corresponding decrease in MgO, Cr, Ni and Co[11]. No similar trend is shown in the selected samples according to the variation diagrams (Fig. S4i-l). The picrites - boninites exhibit relatively high MgO (up to 15.2 wt%, mean 14.6 wt%), Cr (up to 1450 ppm, mean 1238 ppm), Ni (up to 467 ppm, mean 208 ppm) without any sharp negative correlation against that of Zr, Hf or LREE. Therefore, we see no evidence that the Zanhuang magmas underwent significant contamination by interacting with older crust, which is consistent with our inferred derivation from the oceanic mantle in a juvenile forearc setting.

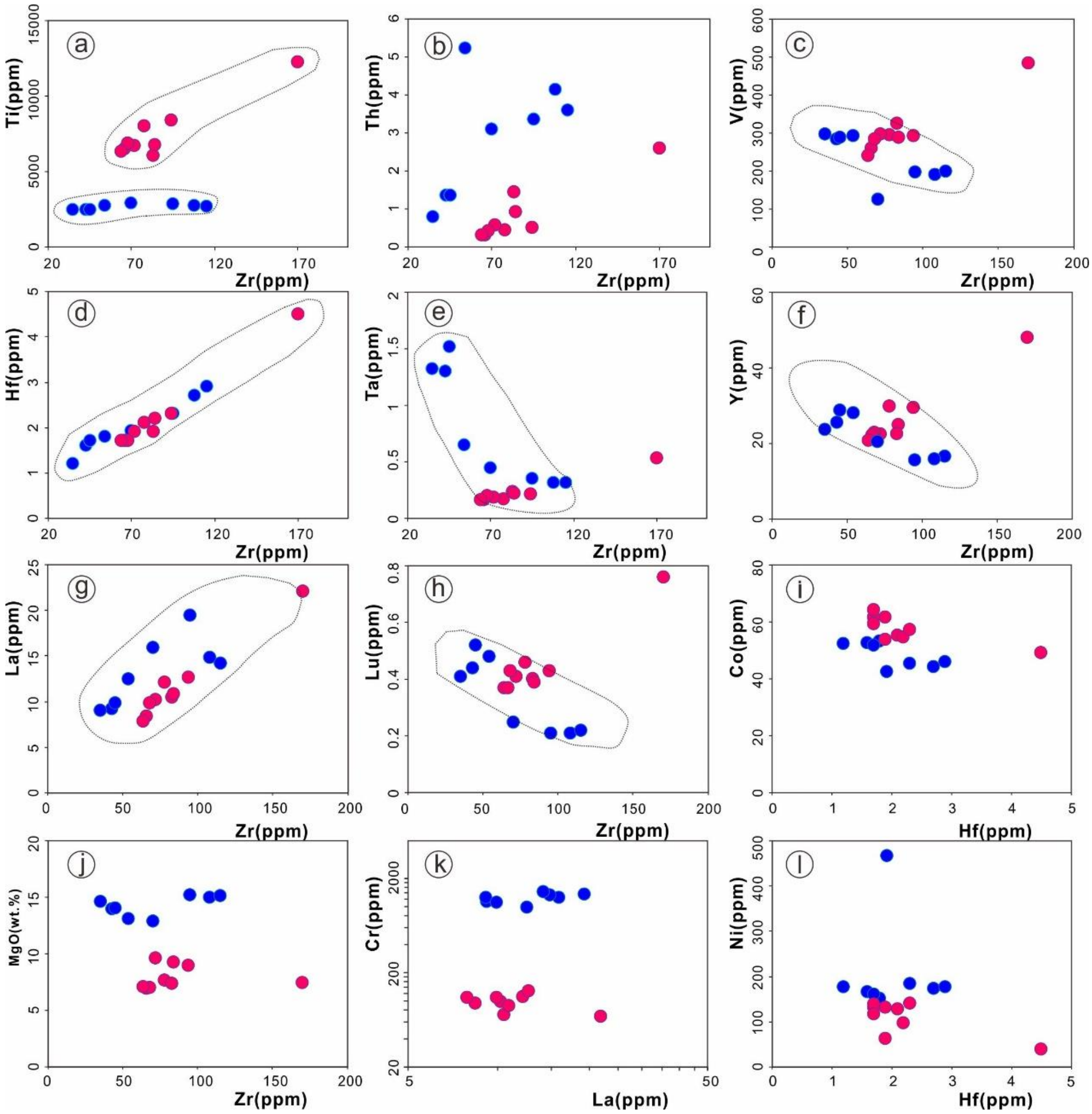


**Fig. S4**: Variation diagrams of Zr versus selected elements for the picritic-boninitic metabasites and tholeiitic metabasalts; blue circles represent picritic-boninitic metabasites, red circles represent tholeiitic metabasalts.

## Supplementary References


1 Hoskin, P. & Black, L. P. Metamorphic zircon formation by solid-state recrystallization of protolith igneous zircon. *Journal of Metamorphic Geology* **18**, 423-439 (2000).

2 Grant, M. L., Wilde, S. A., Wu, F. & Yang, J. The application of zircon cathodoluminescence imaging, Th-U-Pb chemistry and U-Pb ages in interpreting discrete magmatic and high-grade metamorphic events in the North China Craton at the Archean/Proterozoic boundary. *Chemical Geology* **261**, 155-171 (2009).

3 Wu, Y. & Zheng, Y. Genesis of zircon and its constraints on interpretation of U-Pb age. *Chinese Science Bulletin* **49**, 1554-1569 (2004).

4 Corfu, F., Hanchar, J. M., Hoskin, P. & Kinny, P. Atlas of Zircon Textures. *Reviews in Mineralogy & Geochemistry* **53**, 469–500 (2003).

5 Hoskin, P. & Schaltegger, U. The Composition of Zircon and Igneous and Metamorphic Petrogenesis. *Reviews in Mineralogy and Geochemistry* **53**, 27–62 (2003).

6 Vavra, G., Schmid, R. & Gebauer, D. Internal morphology, habit and U-Th-Pb microanalysis of amphibolite-to-granulite facies zircons: geochronology of the Ivrea Zone (Southern Alps). *Contributions to Mineralogy and Petrology* **134**, 380–404 (1999).

7 Rubatto, D. Zircon trace element geochemistry: partitioning with garnet and the link between U-Pb ages and metamorphism. *Chemical Geology* **184**, 123-138 (2002).

8 Whitehouse, M. J. & Platt, J. P. Dating high-grade metamorphism-constraints from rare-earth elements in zircon and garnet. *Contributions to Mineralogy and Petrology* **145**, 61-74 (2003).

9 Humphris, S. E. & Thompson, G. Trace element mobility during hydrothermal alteration of oceanic basalts. Geochimica et Cosmochimica Acta **42**, 127–136 (1978).

10 Polat, A. & Hofmann, A. W. Alteration and geochemical patterns in the 3.7–3.8 Ga Isua greenstone belt, West Greenland. Precambrian Research **126**, 197-218 (2003).

11 Puchtel, I. S., Hofmann, A. W., Mezger, K., Jochum, K. P., Shchipansky, A. A., Samsonow, S. V. Oceanic plateau model for continental crustal growth in the Archaean: A case study from the Kostomuksha greenstone belt, NW Baltic Shield - ScienceDirect. Earth and Planetary Science Letters **155**, 57-74 (1998).
