# Supplementary material for: Alpine-style nappes thrust over ancient North China continental margin demonstrate large Archean horizontal plate motions
Source: Nat Commun. 2021 Oct 26;12:6172. doi: 10.1038/s41467-021-26474-7 (PMC8548327; doi:10.1038/s41467-021-26474-7)
Supplement: Supplementary file 3 — Description of Additional Supplementary Files [file 41467_2021_26474_MOESM3_ESM.pdf]

Description of Additional Supplementary Files

File Name: Supplementary Data 1

Description: LA-ICP-MS zircon U-Pb data and rare earth element data for representative samples.

File Name: Supplementary Data 2

Description: Major and trace element compositions and significant element ratios with sample locations for representative samples.
